# How the UK public views the use of diagnostic decision aids by physicians: a vignette-based experiment

**DOI:** 10.1093/jamia/ocad019

**Published:** 2023-02-16

**Authors:** Martine Nurek, Olga Kostopoulou

**Affiliations:** Department of Surgery and Cancer, Imperial College London, London, UK; Department of Surgery and Cancer, Imperial College London, London, UK

**Keywords:** diagnosis, artificial intelligence, individual differences, decision aids, worry, health regulatory focus, trust in physicians

## Abstract

**Objective:**

Physicians’ low adoption of diagnostic decision aids (DDAs) may be partially due to concerns about patient/public perceptions. We investigated how the UK public views DDA use and factors affecting perceptions.

**Materials and Methods:**

In this online experiment, 730 UK adults were asked to imagine attending a medical appointment where the doctor used a computerized DDA. The DDA recommended a test to rule out serious disease. We varied the test’s *invasiveness*, the doctor’s *adherence* to DDA advice, and the *severity* of the patient’s disease. Before disease severity was revealed, respondents indicated how worried they felt. Both before [t1] and after [t2] severity was revealed, we measured satisfaction with the consultation, likelihood of recommending the doctor, and suggested frequency of DDA use.

**Results:**

At both timepoints, satisfaction and likelihood of recommending the doctor increased when the doctor adhered to DDA advice (*P *≤* *.01), and when the DDA suggested an invasive versus noninvasive test (*P *≤* *.05). The effect of adherence to DDA advice was stronger when participants were worried (*P *≤* *.05), and the disease turned out to be serious (*P *≤* *.01). Most respondents felt that DDAs should be used by doctors “sparingly” (34%_[t1]_/29%_[t2]_), “frequently,” (43%_[t1]_/43%_[t2]_) or “always” (17%_[t1]_/21%_[t2]_).

**Discussion:**

People are more satisfied when doctors adhere to DDA advice, especially when worried, and when it helps to spot serious disease. Having to undergo an invasive test does not appear to dampen satisfaction.

**Conclusion:**

Positive attitudes regarding DDA use and satisfaction with doctors adhering to DDA advice could encourage greater use of DDAs in consultations.

## BACKGROUND

The scientific literature is rife with studies demonstrating people’s reluctance to utilize artificial intelligence (AI) advice,^1–3^ despite its proven superior accuracy in comparison to the unaided human mind.^4–6^ This phenomenon has been observed with lay people and professionals alike in different domains of human judgment and decision-making, and it has been attributed to different psychological mechanisms; for example, confidence in one’s own judgment,^7^ lack of trust in AI,^8^^,^^9^ difficulty in understanding AI advice,^10^ and “uniqueness neglect.”^2^

These mechanisms also hold for physicians and can partly explain the low adoption of computerized clinical decision support in general^11^ and of diagnostic decision aids (DDAs) specifically.^12^ Physicians may not perceive the need for DDA advice, either because they do not recognize cases that appear routine but are in fact hard to diagnose, or because they perceive their personal diagnostic error rate to be too low to justify using a DDA.^12^ They may also be concerned about how patients and colleagues will perceive them if they appear to rely on external support to deliver a diagnosis rather than their own knowledge and proficiency.^13^ Indeed, early research suggested that people judge doctors who use DDAs to be less competent and professional than doctors who rely solely on their own judgment.^14^^,^^15^ In contrast, more recent research found that people appreciate professionals who use decision aids to augment their judgment,^16^ and prefer AI in an advising role over fully automated decision-making.^2^^,^^17^

Eastwood et al^18^ demonstrated that people prefer human experts over a statistical formula—the “human-is-better” effect. They also prefer a decision strategy that evaluates all the information available over one that evaluates only 2 or 3 predictive information items—the “more-is-better” effect. Their study participants believed that human experts (doctors and judges) use fully rational decision-making strategies. However, research has repeatedly shown that experts usually decide based on relatively few information items.^19^ This inconsistency speaks in favor of a combined (human+AI) approach: the AI processes all the available information and provides the human with one or more suggestions, which the human then evaluates according to his/her experience and knowledge before coming to a decision. Palmeira and Spassova^16^ found this combined or hybrid approach to be preferred over both a decision-aid-only approach and an expert-only approach. Thus, it appears that people are intuitively aware of the benefits of using AI but wish for the human expert to remain in the loop and not be replaced entirely by AI.^17^

In a study by Pezzo and Pezzo,^20^ psychology undergraduates read different versions of a hypothetical scenario of a medical diagnosis. In one experimental condition, a DDA was available, described as being correct more frequently than doctors in general. When the doctor followed the advice of the DDA but missed the diagnosis, harming the patient as a result, he/she was judged to be significantly less negligent than a control condition, which did not mention a DDA—even though the doctor was always described as thorough, one who “*considers several possible causes … and orders a series of tests*” (p. 50). Interestingly, in the scenario version where the doctor followed DDA advice and diagnosed the condition correctly, the quality of his/her decision was judged as lower than control. As the authors suggest, “*it appears that people may give a decision aid some of the credit for a positive outcome and some of the blame for a negative outcome*” (p. 51). In a follow-up experiment with medical and nonmedical students as participants, a doctor who made a diagnostic error resulting in a patient’s death was judged as more at fault if he/she had defied the DDA’s advice than if he/she had followed this advice. Thus, it appears that following the advice of a DDA may have a protective effect in case of patient harm and litigation.

Recently, Pezzo et al^21^ studied the effects of not consulting or not heeding the advice of a DDA that suggested the most likely diagnosis. They used a scenario with a negative patient outcome: life-changing complications following a misdiagnosis of a serious condition. Participants (university students) were asked to imagine that they were jurors in a medical malpractice trial. When the DDA was available and consulted, the physician either followed or defied its suggestion. When followed, the suggestion was set to be incorrect; when defied, it was set to be correct. When the physician followed the (incorrect) suggestion, he/she was rated more positively than control (no DDA was mentioned), and when he/she defied the suggestion. Thus, as in the studies by Pezzo and Pezzo^20^ and Arkes et al,^14^ following the incorrect advice of a DDA had a protective effect, presumably by removing some of the physician’s culpability for the patient’s poor outcome.

None of the previous studies explored how worry might impact perceptions of DDA use. Worry influences risk perception, which, in turn, influences intentions to engage in health-protective behaviors.^22^ Furthermore, Peters et al^23^ found that worry about medical errors predicted intentions to take precautionary actions against such errors and that worry was a stronger predictor than risk perception. Thus, people who worry more about the (small) probability of a serious disease may be more accepting of interventions aimed at detecting the disease.

The study reported here aimed to test further the boundaries of the public’s perception about doctors who use computerized diagnostic aids. It extends Pezzo and colleague’s findings in several ways:

1. In addition to manipulating the physician’s adherence to DDA advice, we also manipulated the severity of the patient’s actual disease and the invasiveness of the diagnostic test that was recommended by the DDA, expecting them to be important factors shaping responses.
2. We elicited responses at 2 points in time—both before and after the patient’s actual disease were revealed.
3. We tested whether worry moderated responses, by measuring how worried respondents thought that they would be in the hypothetical situation.
4. We recruited a wide range of respondents (rather than university students only), representative of the UK population in terms of age, sex, and ethnicity.

We described the DDA as a differential diagnosis generator, providing the physician with a list of possible diagnoses to consider, and recommending a specific test to rule out a serious disease. We measured respondents’ satisfaction with the consultation, likelihood of recommending the physician to a family member, and suggested frequency of DDA use in the consultation. Prior to data collection, we preregistered the following hypotheses on the Open Science Framework (preregistration DOI: 10.17605/OSF.IO/NU862):

**H1—Physician adherence to DDA advice:** Based on previous studies,^16^^,^^17^^,^^21^ we expected physician adherence to lead to more favorable responses than nonadherence.

**H2—Test invasiveness:** We expected the invasive test to lead to less favorable responses than the noninvasive test. This hypothesis relied on plausibility rather than theory: respondents’ expectations of the unpleasant and stressful experience of the invasive test would lower their ratings.

**H3—Disease severity**: Extrapolating from the findings of Pezzo and Pezzo,^20^ we expected the outcome (serious vs nonserious disease) to moderate the impact of adherence and test invasiveness. Specifically, we expected respondents to be more grateful for the doctor’s adherence to DDA advice if it led to detecting the serious disease. Likewise, we expected respondents to be more tolerant of an invasive test if enabled detecting the serious disease. We thus expected the following interactions:

**H3_a_—Severity-by-invasiveness interaction:** a serious disease would reduce the negative impact of test invasiveness since the test led to diagnosing the disease.

**H3_b_—Severity-by-adherence interaction:** a serious disease would increase the positive effect of physician adherence since adherence led to diagnosing the disease.

## MATERIALS AND METHODS

### Participants

#### Sample size calculation

We calculated sample size using the G*Power software (v. 3.1.9.4). We estimated that with 720 respondents, we would have 90% power to detect a small effect (*f*^2^=0.02) in a multiple linear regression with 3 predictors (adherence to DDA advice, test invasiveness, disease severity) and their interactions.

#### Recruitment

English-speaking adults residing in the United Kingdom were eligible to take part. Recruitment was carried out by Qualtrics, who source participants via opt-in market research panels. Qualtrics ensured that the sample was representative of the UK population in terms of age, sex, and ethnicity.

### Materials

We constructed a scenario describing a patient consulting a family physician for stomach pain and constipation. After taking the patient’s history and conducting a physical examination, the physician consults a “NHS-approved computer program.” The physician explains to the patient that the DDA has suggested a number of possible diagnoses; most are benign, but one is serious. The physician also explains that the DDA recommends a follow-up test to rule out the serious disease. The remainder of the scenario varied in a 2 (adherence)×2 (invasiveness)×2 (severity) between-participant design. The 8 versions of this scenario can be found in the Supplementary Material S1.

### Procedure

Participants completed an online questionnaire presented on the Qualtrics platform. After providing informed electronic consent (by ticking a box labeled “I agree to participate”), they filled in demographic information (age, gender, ethnicity, education level; see Supplementary Material S2). They were randomly assigned to view 1 of the 8 scenario versions. Measures were taken at 2 timepoints: before the patient’s actual disease was revealed (timepoint 1—t1) and after it was revealed (timepoint 2—t2). At t1, participants responded to questions 1 to 5 below. At t2, they responded again to questions 2 to 5—that is, worry was not measured at t2.

1. “*To what extent are you worried that your symptoms might be caused by something serious?*” Participants responded on a 4-point scale: “not at all worried” (1), “a little bit worried” (2), “moderately worried” (3), and “very worried” (4).
2. “*How satisfied are you with how this doctor has handled your problem?*” Participants responded on a 0–100 scale, anchored at “not satisfied” (0) and “very satisfied” (100).
3. “*Would you recommend this doctor to a family member?*” Participants responded on a 1–4 scale: “definitely not” (1), “probably not” (2), “probably would” (3), and “definitely would” (4).
4. “*Please rate your overall satisfaction with the consultation.*” Participants responded on a 0–100 scale, anchored at “not satisfied” (0) and “very satisfied” (100).
5. “*In your view, how often should doctors use computer programs (like the one used here)?*” Participants responded on a 1–4 scale: “never” (1), “sparingly (less than half of the time)” (2), “frequently (most of the time)” (3), and “always” (4).

Following responses to the scenario, participants completed 3 measures of individual differences:

1. the 11-item Trust in Physicians scale, which measures the extent to which patients trust their doctor’s knowledge, advice, and care^24^;
2. the 12-item Health Regulatory Focus questionnaire assessing tendencies to avoid negative health outcomes (“prevention focus,” Cronbach’s α≥0.75) and/or attain positive health outcomes (“promotion focus,” Cronbach’s α≥0.68, see Supplementary Material S4)^25^; and
3. the single-item Maximizer-Minimizer Elicitation question (MM1), measuring strength of preference for an active versus passive (“wait-and-see”) approach to healthcare (see Supplementary Material S5).^26^

The order of these 3 scales was randomized per participant.

Finally, participants were asked to provide some health-related information (see Supplementary Material S6), including their physical fitness relative to the average person of their age (*less fit, equally fit, more fit*), chronic illness (*present, absent*), and the number of physician consultations in the past year (*0–4, 5–9, 10-14, 15–19, 20+*). They were also asked to indicate why, in their view, physicians might use computers during consultations. They were offered the following options and they could tick all that applied:

- *For reimbursement purposes*
- *To make sure they record my information correctly and completely*
- *To make sure that they remember what happened in my past consultations*
- *To make sure that they do not miss anything, for example, screening tests that are due*
- *To avoid talking directly to me*
- *To sell my data to pharmaceutical companies*
- *Other, please explain. [textbox]*

**Figure 1.**
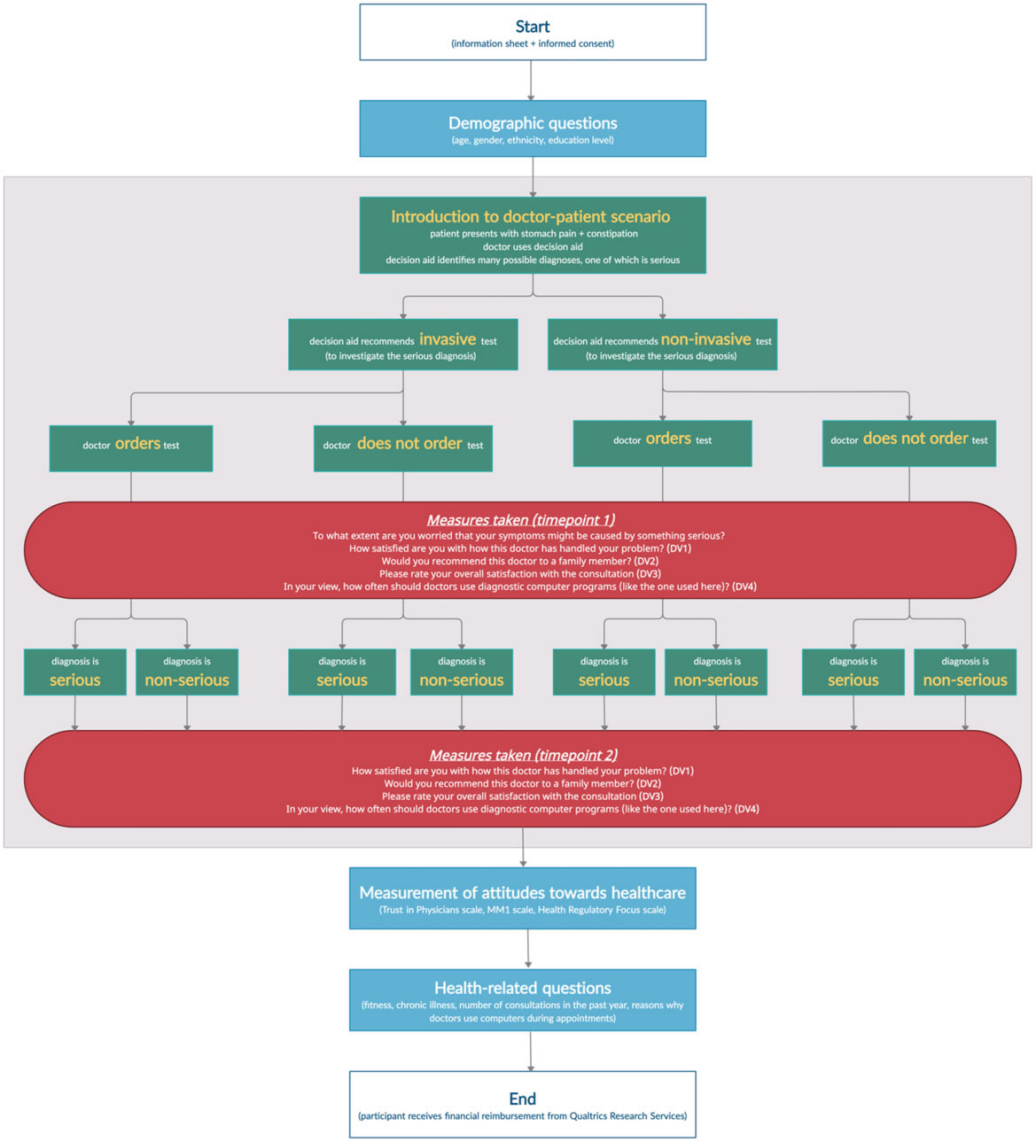
Graphical representation of the study procedure.

The study procedure is displayed graphically in Figure 1. Qualtrics applied a speed check, by measuring the median time taken by the first 10% of respondents (4.5 min), and then excluding and replacing any respondent that took less than half of the median time.

### Analyses

The analyses were preregistered on the Open Science Framework. We obtained responses on 4 rating scales measuring satisfaction with the physician, likelihood of recommending the physician to a family member, satisfaction with the consultation, and suggested frequency of DDA use. We thus aimed to construct 4 separate regression models, one for each measure, and apply a Bonferroni adjustment to the threshold for statistical significance.

Final responses, that is, those taken at t2, were regressed upon the 3 manipulated factors (invasiveness, adherence, severity) and their interactions in separate multiple linear regression models. The same models (excluding severity) were repeated for interim responses, that is, those taken at t1.

Anticipating that worry would moderate the effects of the manipulated factors, we then repeated these models including 3 interactions with adherence to DDA advice, test invasiveness, and disease severity. The effects of the individual difference measures, state of health, and demographic variables are reported in Supplementary Material S7.

## RESULTS

Responses of 730 participants were analyzed. 263 participants failed the speed check and were automatically replaced by Qualtrics by recruiting more respondents until the required sample size was reached. The final sample was not representative of the population in terms of education level (skewed towards postgraduate education), due to an error by Qualtrics. Respondents were reimbursed for their time. The amount of compensation was determined by Qualtrics with no involvement from the researchers. Table 1 presents the characteristics of the final sample (*N *=* *730).

**Table 1.** Sample characteristics

Age	*M* = 45.3 years, SD = 15.8 (range 18–91 years)	
Sex	Male	50.3% (367/730)
	Female	49.5% (361/730)
	Nonbinary	0.1% (1/730)
	Prefer not to say	0.1% (1/730)
Ethnicity	White	88% (640/730)
	Asian/Asian British	6% (41/730)
	Black/African/Caribbean/Black British	4% (27/730)
	Mixed ethnic descent	2% (17/730)
	Other ethnic descent	1% (5/730)
Education level	Postgraduate degree	40% (289/730)
	Undergraduate degree	41% (301/730)
	High school	18% (133/730)
	Less than high school	1% (7/730)

Two of the 4 dependent variables (*satisfaction with the doctor* and *satisfaction with the consultation*) were conceptually related, measured on the same scale (0–100), and displayed the same pattern of results. We therefore merged them by taking the mean per participant per timepoint. The new variable is simply termed *satisfaction*. Results are presented separately for the 2 original variables (*satisfaction with the doctor* and *satisfaction with the consultation*) in Supplementary Material S8 and S9.

We adjusted the threshold of significance for the 3 dependent variables (satisfaction, recommendation, DDA use) by dividing 0.05 by 3, which gave us a new Bonferroni-adjusted threshold of *P *≤* *.017. (NB. The preregistration expected a Bonferroni-adjusted threshold of *P *≤* *.0125, that is, for 4 dependent variables; results do not change when we apply this threshold.)

Table 2 presents descriptive statistics for the 3 dependent variables at each timepoint. Mean satisfaction was above average (>50 on the 0–100 scale). Almost half of the sample indicated that they would “probably” recommend the doctor to a family member. Most respondents indicated that DDAs should be used in clinical practice, be it “sparingly,” “frequently,” or “always,” with most opting for “frequently.” A small minority felt that they should never be used. This minority was slightly younger than the sample average (mean age 39.4 vs 45.3) and included more females than the whole sample (64% vs 50%). A larger minority responded that DDAs should always be used. Their mean age reflected the broader sample (46.2 vs 45.3), and there were more males (60% vs 50%).

**Table 2.** Descriptive statistics for the dependent variables (satisfaction, recommendation, and DDA use) at timepoints 1 and 2

		Timepoint 1	Timepoint 2
Satisfaction		*M *=* *66.8 (SD* *=* *25.4)	*M *=* *68.3 (SD* *=* *27.7)
Likelihood of recommending the doctor	“definitely not”	51 (7%)	70 (9.6%)
	“probably not”	157 (21.5%)	125 (17.1%)
	“probably would”	354 (48.5%)	333 (45.6%)
	“definitely would”	168 (23%)	202 (27.7%)
Suggested frequency of DDA use	“never”	46 (6.3%)	55 (7.5%)
	“sparingly (less than half of the time)”	254 (33.6%)	213 (29.2%)
	“frequently (most of the time)”	316 (43.3%)	311 (42.6%)
	“always”	123 (16.8%)	151 (20.7%)

Table 3 displays the results of the regression models used to test the effect of the manipulated factors (adherence, invasiveness, severity) at each timepoint. In accordance with H1, physician adherence to DDA advice led to higher satisfaction and likelihood of recommendation at both timepoints. Contrary to H2, satisfaction and likelihood of recommendation were higher following the DDA suggesting an invasive versus noninvasive test. We detected no significant impact of any of the factors on opinions about frequency of DDA use.

**Table 3.** Effects of the manipulated factors and their interactions at timepoints 1 and 2

	Timepoint 1			Timepoint 2		
	Satisfaction	Recommendation	DDA use	Satisfaction	Recommendation	DDA use
	*b* [95% CI]	*b* [95% CI]	*b* [95% CI]	*b* [95% CI]	*b* [95% CI]	*b* [95% CI]
	*P*	*P*	*P*	*P*	*P*	*P*
Adherence	20.18 [15.29, 25.07] *P < *.001	0.63 [0.46, 0.79] *P < *.001	0.05 [−0.12, 0.22] *P *=* *.538	17.16 [10.02, 24.30] *P < *.001	0.50 [0.27, 0.73] *P < *.001	0.04 [−0.21, 0.28] *P *=* *.766
Invasiveness	8.88 [3.95, 13.82] *P < *.001	0.22 [0.06, 0.39] *P *=* *.009	−0.10 [−0.27, 0.07] *P *=* *.232	9.26 [1.93, 16.60] *P *=* *.013	0.24 [−0.00, 0.48] *P *=* *.051	−0.03 [−0.28, 0.22] *P *=* *.816
Severity	—	—	—	−16.37 [−23.68, −9.05] *P < *.001	−0.60 [−0.84, −0.37] *P < *.001	−0.12 [−0.37, 0.13] *P *=* *.357
Severity×Adherence	—	—	—	13.75 [3.42, 24.07] *P *=* *.009	0.54 [0.21, 0.88] *P *=* *.002	0.35 [−0.00, 0.70] *P *=* *.052
Severity×Invasiveness	—	—	—	0.42 [−9.98, 10.82] *P *=* *.937	0.02 [−0.32, 0.36] *P *=* *.912	0.03 [−0.32, 0.39] *P *=* *.848
Adherence×Invasiveness	−6.02 [−12.91, 0.88] *P *=* *.087	−0.15 [−0.39, 0.08] *P *=* *.191	0.16 [−0.07, 0.40] *P *=* *.176	−8.54 [−18.73, 1.66] *P *=* *.101	−0.25 [−0.59, 0.08] *P *=* *.133	0.14 [−0.21, 0.49] *P *=* *.429
Adherence×Invasiveness×Severity	—	—	—	3.19 [−11.36, 17.73] *P *=* *.667	0.17 [−0.30, 0.64] *P *=* *.478	0.01 [−0.49, 0.50] *P *=* *.982

*Note*: Cells contain regression coefficients (*b*), 95% confidence intervals (CI), and *P* values. Invasiveness, Adherence, and Severity were coded 0 = not invasive/no adherence/not serious, 1 = invasive/adherence/serious. The Bonferroni-adjusted threshold of significance is *P *≤* *.017.

**Figure 2.**
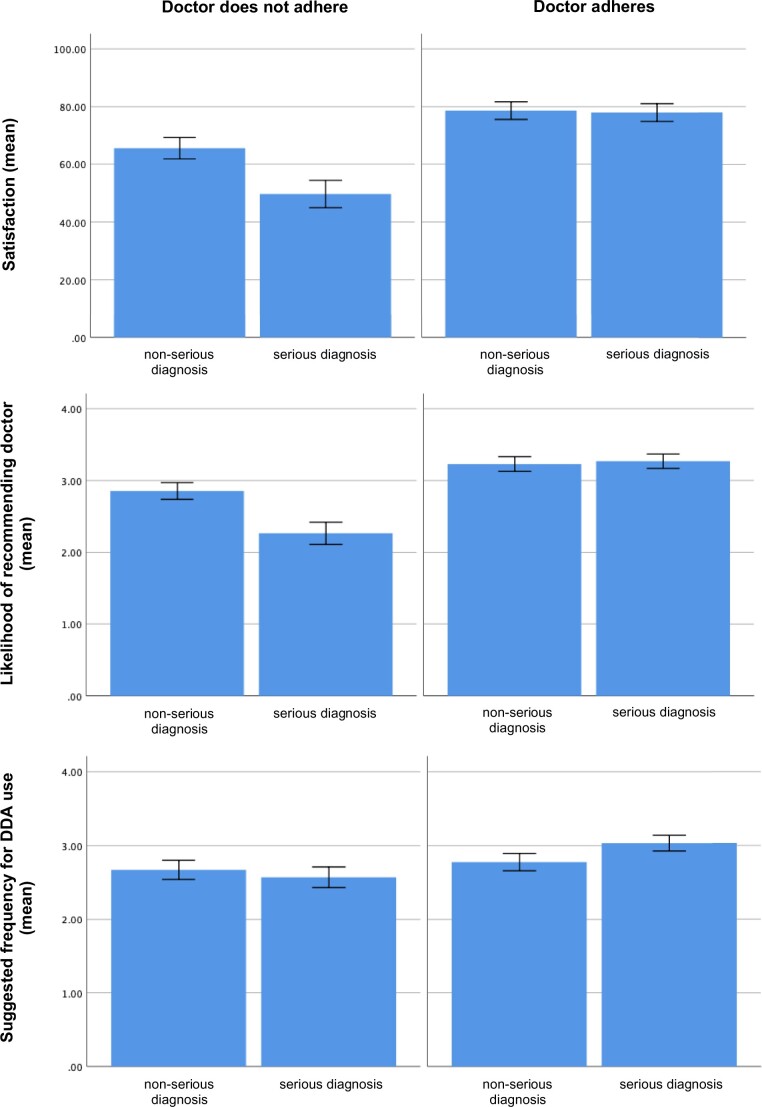
Effect of severity of diagnosis on responses (satisfaction, recommendation, DDA use) by adherence to DDA. Error bars display 95% CIs. CIs: confidence intervals; DDA: diagnostic decision aid.

We found no evidence for H3_a_: severity of the patient’s disease did not moderate the effect of test invasiveness. We did however find evidence in support of H3_b_: severity moderated the effect of adherence. Figure 2 shows that when the doctor adhered to DDA advice, satisfaction and likelihood of recommendation were high irrespective of severity. However, when the doctor did not adhere and the patient’s disease turned out to be serious, both satisfaction and recommendation were lower than in the nonserious disease. A similar trend was identified for suggested frequency of DDA use, but this narrowly missed significance (*P *=* *.052). No other interactions were significant.

**Figure 3.**
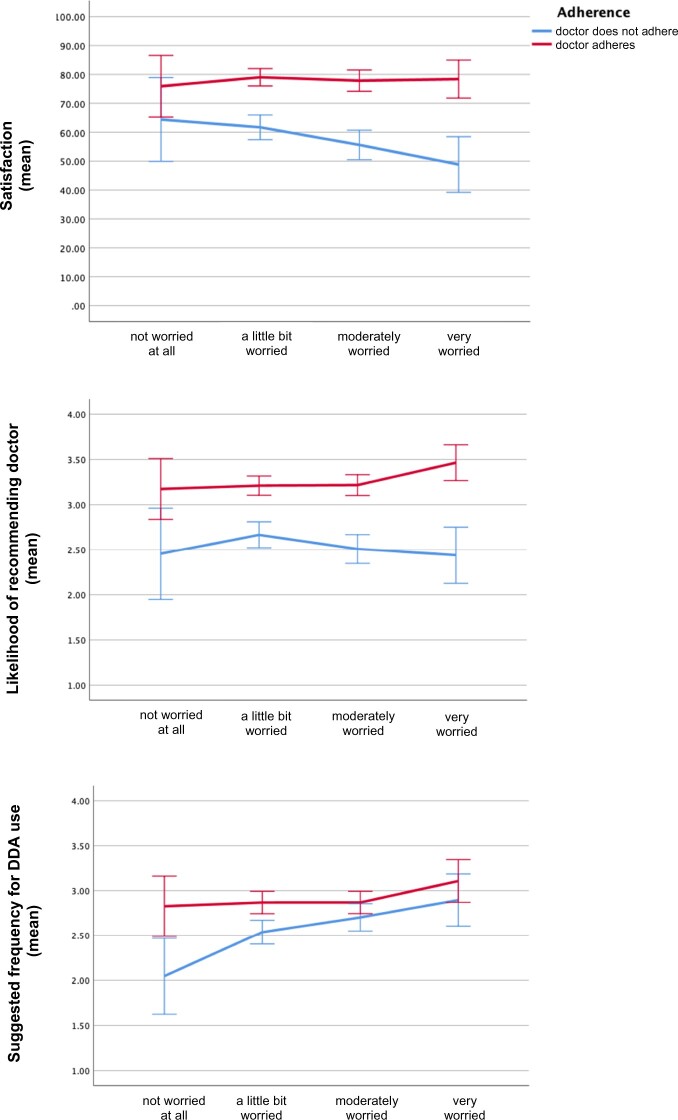
Effect of doctor’s adherence on the dependent variables (measured at timepoint 2) by level of worry (measured at timepoint 1). Error bars display 95% CIs. CIs: confidence intervals.

Table 4 displays the results of the regression models that included worry and its interaction with the manipulated factors. As in the earlier models, there were significant adherence-by-severity interactions for all dependent variables. Furthermore, we detected significant adherence-by-worry interactions for satisfaction and recommendation at both timepoints. As worry increased, satisfaction with the nonadherent doctor decreased, while satisfaction with the adherent doctor was immune to worry (Figure 3, top graph). The adherent doctor was more likely to be recommended than the nonadherent doctor and this difference was more pronounced for those who were either moderately or very worried (Figure 3, middle graph). Finally, as worry increased, so did the suggested frequency of DDA use, especially when the doctor had not adhered to DDA advice (Figure 3, bottom graph). However, we are stating this last interaction with caution, as it was only significant at t2 and may be driven by the small subgroup of the “not worried at all” (6.2% of the sample).

**Table 4.** Effects of the manipulated factors and interactions with worry (timepoint 1) on responses at timepoints 1 and 2

	Timepoint 1			Timepoint 2		
	Satisfaction	Recommendation	DDA use	Satisfaction	Recommendation	DDA use
	*b* [95% CI]	*b* [95% CI]	*b* [95% CI]	*b* [95% CI]	*b* [95% CI]	*b* [95% CI]
	*P*	*P*	*P*	*P*	*P*	*P*
Adherence	1.13 [−10.55, 12.80] *P *=* *.850	0.04 [−0.36, 0.43] *P *=* *.853	0.16 [−0.25, 0.56] *P *=* *.450	2.82 [−10.48, 16.12] *P *=* *.677	0.11 [−0.33, 0.55] *P *=* *.620	0.45 [0.00, 0.90] *P *=* *.050
Invasiveness	−1.82 [−13.71, 10.08] *P *=* *.765	−0.14 [−0.54, 0.26] *P *=* *.503	−0.31 [−0.72, 0.10] *P *=* *.142	5.89 [−7.80, 19.58] *P *=* *.399	0.19 [−0.26, 0.64] *P *=* *.409	−0.19 [−0.66, 0.27] *P *=* *.413
Severity	—	—	—	−18.23 [−32.00, −4.46] *P *=* *.010	−0.55 [−1.00, −0.10] *P *=* *.018	−0.23 [−0.70, 0.24] *P *=* *.333
Adherence×Severity	—	—	—	13.58 [3.29, 23.87] *P *=* *.010	0.53 [0.20, 0.87] *P *=* *.002	0.36 [0.02, 0.71] *P *=* *.041
Invasiveness×Severity	—	—	—	−0.25 [−10.60, 10.11] *P *=* *.963	0.01 [−0.33, 0.35] *P *=* *.962	0.07 [−0.28, 0.42] *P *=* *.704
Adherence×Invasiveness	−5.41 [−12.23, 1.41] *P *=* *.120	−0.15 [−0.38, 0.08] *P *=* *.202	0.12 [−0.12, 0.35] *P *=* *.320	−8.27 [−18.44, 1.89] *P *=* *.110	−0.27 [−0.60, 0.06] *P *=* *.111	0.13 [−0.21, 0.48] *P *=* *.453
Adherence×Invasiveness×Severity	—	—	—	3.64 [−10.87, 18.16] *P *=* *.623	−0.18 [−0.29, 0.66] *P *=* *.450	−0.04 [−0.53, 0.45] *P *=* *.873
Worry (timepoint 1)	−8.90 [−12.51, −5.30] *P *<* *.001	−0.23 [−0.35, −0.11] *P *<* *.001	0.14 [0.02, 0.27] *P *=* *.025	−6.72 [−11.17, −2.27] *P *=* *.003	−0.07 [−0.22, 0.07] *P *=* *.335	0.17 [0.02, 0.32] *P *=* *.026
Worry×Adherence	7.09 [2.93, 11.25] *P *=* *.001	0.22 [0.08, 0.36] *P *=* *.002	−0.03 [−0.18, 0.11] *P *=* *.666	5.40 [0.97, 9.82] *P *=* *.017	0.15 [0.01, 0.30] *P *=* *.039	−0.16 [−0.31, −0.01] *P *=* *.040
Worry×Invasiveness	3.90 [−0.26, 8.07] *P *=* *.066	0.13 [−0.01, 0.27] *P *=* *.064	0.08 [−0.06, 0.23] *P *=* *.254	1.27 [−3.17, 5.70] *P *=* *.575	0.02 [−0.13, 0.16] *P *=* *.801	0.06 [−0.09, 0.21] *P *=* *.406
Worry×Severity	—	—	—	0.83 [−3.60, 5.25] *P *=* *.714	−0.02 [−0.16, 0.13] *P *=* *.791	0.04 [−0.11, 0.19] *P *=* *.607

*Note*: Cells contain regression coefficients (*b*), 95% confidence intervals (CI), and *P* values. Worry was coded 1 = not at all worried, 2 = a little bit worried, 3 = moderately worried, 4 = very worried. Worry was measured only at timepoint 1.

When we added measures of individual differences to the model, we found that greater health regulatory focus (HRF) and trust in physicians (TIP) were both associated with significantly more favorable ratings on all dependent variables (satisfaction, recommendation, and DDA use—see Supplementary Material S7).

Finally, we had asked participants to select reasons why doctors might use computers during consultations. Their selections suggested a generally positive attitude towards computer use in the consultation, because it was perceived to ensure that doctors: (1) keep a complete and accurate record of information (70.5%), (2) remember what happened in past consultations (61.4%), and (3) do not miss anything, for example, screening tests that are due (57%) (Supplementary Material S10).

## DISCUSSION

We conducted a study of public attitudes towards the use of computerized DDAs in a primary care setting. Consistent with our hypotheses and prior research,^20^^,^^21^ doctor’s adherence to DDA advice led to greater satisfaction and likelihood of recommending the doctor than nonadherence. Earlier research that produced different findings, namely that “patients derogate physicians who use a DDA,”^14^^,^^15^ used different hypothetical situations and dependent variables to study perceptions. In Arkes et al,^14^ the physician who diagnosed with the help of the aid produced the same diagnosis as the one who diagnosed unaided. Thus, the latter was rated as a better diagnostician. We did not ask participants to rate the physician’s diagnostic acumen and we cannot assume that their recommendation ratings amounted to the same thing. It is more likely that our participants were rating the physician’s thoroughness and safety of care.

Pezzo and Pezzo^20^ found that adherence to DDA advice was viewed more favorably than control (no DDA was mentioned) when the outcome was negative (missed diagnosis leading to reduced quality of life). This is consistent with our finding that adherence to DDA advice led to positive ratings. Pezzo and Pezzo^20^ also found, however, that adherent doctors were viewed *less* favorably than control when the outcome was positive (correct diagnosis leading to patient recovery). This is inconsistent with the present study, where the effect of adherence was positive irrespective of the outcome. These different findings could be due to several subtle differences between the 2 studies. In Pezzo and Pezzo’s study, the serious diagnosis was missed because the doctor adhered to DDA. In our study, the serious diagnosis was missed because the doctor did not adhere to DDA. In Pezzo and Pezzo, the doctor’s actions had serious consequences for the patient (considerable decrease in quality of life). In our study, patient outcome did not depend on the doctor’s actions; even when the serious disease was missed, the patient suffered no material harm, because the disease was promptly diagnosed via a different route. Finally, the nature of DDA advice differed between studies. Pezzo and Pezzo focused on respondents’ reactions to an incorrect/correct diagnosis, delivered with/without assistance from a DDA. We focused on respondents’ willingness to accept an additional diagnostic test suggested by a DDA, which aimed to exclude an unlikely but potentially serious disease. Thus, participants in the 2 studies were responding to very different situations.

The effect of doctor’s adherence to DDA advice was moderated by worry. Except for a small subgroup who were “not worried at all,” most respondents indicated some degree of worry and rated the nonadherent doctor less favorably than the adherent doctor, which became more apparent as worry increased. More worry also appears associated with respondents recommending more frequent DDA use in the consultation, though this deserves to be explored further. To the extent that DDAs are thought to guard against medical errors, patients who worry more about medical errors may also value the use of DDAs in the consultation.^23^

We had expected less favorable ratings when the DDA recommended an invasive than a noninvasive test, though less so when the disease turned out to be serious (ie, a severity-by-invasiveness interaction). In contrast to our hypotheses, ratings were more favorable when the DDA recommended an invasive test, irrespective of disease severity. Having undergone an invasive and risky test to exclude a serious but unlikely disease did not seem to reduce appreciation of the doctor and the consultation in those who turned out to have a nonserious condition. We could hypothesize that respondents perceived the invasive test as more accurate than the noninvasive one and felt that the doctor took their health issue more seriously, carefully weighing risk and benefits. Given that the invasiveness effect was neither theoretically driven nor strong, we cannot draw any firm conclusions. At most, we can claim that we found no evidence to suggest that following DDA advice to order an invasive test leads to negative perceptions.

Our findings suggest that people prefer doctors to heed the advice of computerized DDAs and support their frequent use in practice, especially when they are worried about their health, and when they help doctors not to miss a serious disease. These findings may encourage doctors to use such aids and include patients in the process. A recent systematic review of patient and public attitudes towards clinical AI also found that perceptions were largely favorable, especially in relation to doctors using AI to aid diagnosis, with patients desiring greater education about the use of AI and greater involvement in its development and implementation.^17^ As more and more DDAs are being developed and the public becomes more accustomed to their use in the consultation, it is important to track how perceptions and attitudes change over time, and any individual differences that could explain variability in attitudes.

### Limitations

We used a written scenario to study people’s responses to a hypothetical situation, which, in real life, might have induced stronger feelings to a patient. For example, patients who are not particularly worried about their symptoms but undergo a painful colonoscopy after a DDA recommended it, only to be told that there is nothing to be worried about, might feel less satisfied than our respondents. Future research could explore how real patients feel after such an experience.

We recruited a large sample of the UK adults representative in terms of age, sex, and ethnicity. This differentiates our study from most in the field, which tend to sample from the student population. Our sample was however predominantly white and privileged, which is a problem with this literature, as noted in a recent systematic review.^17^ Greater diversity is needed if we are to the develop equitable AI solutions that address the needs and concerns of all stakeholders.

Our sample was also skewed towards postgraduates, who are likely more familiar with clinical AI than less-educated populations.^27^ We found little evidence to suggest that this consistently influenced responses, but we acknowledge that it limits the generalizability of our findings. We are also aware that AI perceptions are sensitive to social and cultural norms,^28^ therefore generalizability beyond the UK population could be questioned. Furthermore, we did not explore the role of participants’ familiarity with clinical AI, given the number of patient-facing symptom checkers currently available. Respondents who engage with symptom checkers or other types of computerized clinical decision aids may have different perceptions of their use in the consultation compared to patients who do not use them.^29^ Generalizability may also be limited by our use of a single hypothetical scenario. Although a previous study found no effect of symptom severity on evaluations of doctors using a DDA,^14^ the generalizability of our findings to other clinical presentations requires further testing.

Our study could benefit from some qualitative data to inform our findings. For example, exploring how respondents felt about having to undergo an invasive test—and their past experiences of such tests—could shed light to the counterintuitive finding that invasiveness appeared to increase satisfaction. Furthermore, in neither case of nonadherence in our study did the patient suffer a harmful outcome. We suspect that nonadherence indicated to our participants lack of professionalism and thoroughness and was therefore judged harshly, as people would expect their doctor to investigate a potentially sinister cause. Qualitative data could provide evidence for this hypothesis.

Finally, although we described the DDA in our study as “NHS-approved,” we did not specify its accuracy. “Studies that exclude accuracy information may unfairly bias their findings against DDA acceptance.”^21^ It is therefore noteworthy that DDA perceptions were as positive as they were in the present study, which may in fact *under*estimate peoples’ true enthusiasm for DDAs.

## CONCLUSION

People hold largely positive attitudes towards the use of computerized DDAs in the clinical consultation. They view positively doctors who heed their advice and are not less satisfied if they are asked to undergo an invasive test that the DDA recommended in order to rule out a serious but unlikely disease. Doctors need not feel that they are thought of less positively by the public for using DDAs.

## Supplementary Material

ocad019_Supplementary_Data

## Data Availability

The data are publicly available on the OSF: https://osf.io/mprfy/files/osfstorage/63e68cb6cb544b01179e6191.
