# Supplementary material for: How the UK public views the use of diagnostic decision aids by physicians: a vignette-based experiment
Source: J Am Med Inform Assoc. 2023 Feb 16;30(5):888–98. doi: 10.1093/jamia/ocad019 (PMC10114121; doi:10.1093/jamia/ocad019)
Supplement: ocad019_Supplementary_Data [file ocad019_supplementary_data.docx]

**SM1: The eight versions of the clinical scenario**

| **Introduction**  (seen by all) | **Please imagine that you have been experiencing stomach pain and constipation on-and-off for about a month.**  At first, you thought the pain was due to irritable bowel syndrome (IBS), which you were diagnosed with two years ago. (IBS is a common and benign condition, affecting about 17% of the population. It affects the digestive system, causing symptoms like stomach cramps, bloating, diarrhoea and constipation. These tend to come and go over time, and can last for days, weeks or months. There is no cure, but diet changes and some medication can help control the symptoms.)  On this occasion, your usual over-the-counter medications aren’t helping, so you decide to make an appointment to see the GP.  During the consultation, the doctor asks several questions about your symptoms and examines your stomach.  Doctor: “I think that your symptoms are due to a flare-up of your IBS. However, I’d like to consult a new, NHS-approved computer program that we are using in the practice. The computer program will provide me with a list of possible diagnoses to consider, based on your symptoms. This helps me to make sure that I haven’t missed anything.” |
| --- | --- |
| **Version 1:**  not invasive,  no adherence,  non-serious | *[timepoint 1 starts here]*  The doctor takes a moment to consult the computer program.  Doctor: “It’s as I suspected: IBS is by far the most likely cause of your symptoms. However, the computer program has flagged some alternative possibilities as well. Most are benign but one is potentially serious. The computer program suggests that we do a stool sample test, to rule out the possibility of a serious cause. This would involve collecting a small amount of poo in a pot (you can do this yourself at home), which we would send to the lab for analysis. However, as I said, IBS is far more likely, so I don’t think that a stool test is necessary at this stage. I’ll spare you the hassle – it’s always an option down the line, if need be.” So, the doctor does not order the stool test but gives you some dietary advice, prescribes medication for the pain and constipation, and tells you to come back in 2 weeks if they continue. You thank the doctor and leave the consultation room.  *[timepoint 1 measures taken here]*  *[timepoint 2 starts here]*  Please imagine that this is what happens after your GP appointment: You make the dietary changes that the doctor suggested and take the medication prescribed. The pain and constipation persist for about a week. At the start of the second week, they begin to subside. They resolve completely by the end of the second week, so you have no need to contact the GP*.*  *[timepoint 2 measures taken here]* |
| **Version 2:**  invasive,  no adherence,  non-serious | *[timepoint 1 starts here]*  The doctor takes a moment to consult the computer program.  Doctor: “It’s as I suspected: IBS is by far the most likely cause of your symptoms. However, the computer program has flagged some alternative possibilities as well. Most are benign but one is potentially serious. The computer program suggests that we do a colonoscopy, to investigate a potentially serious cause. A colonoscopy is a hospital procedure, whereby a thin tube (with a camera inside) is passed into your bottom to look for abnormalities*.* This can be very uncomfortable and there are some risks involved. For example, you may experience stomach cramps and anal bleeding after the procedure. In rare cases, you could get a small tear in your bowels, which may require surgery. However, as I said, IBS is far more likely, so I don’t think that a colonoscopy is necessary at this stage. I’ll spare you the hassle – it’s always an option down the line, if need be.” So, the doctor does not order the colonoscopy but gives you some dietary advice, prescribes medication for the pain and constipation, and tells you to come back in 2 weeks if they continue. You thank the doctor and leave the consultation room.  *[timepoint 1 measures taken here]*  *[timepoint 2 starts here]*  Please imagine that this is what happens after your GP appointment: You make the dietary changes that the doctor suggested and take the medication prescribed. The pain and constipation persist for about a week. At the start of the second week, they begin to subside. They resolve completely by the end of the second week, so you have no need to contact the GP*.*  *[timepoint 2 measures taken here]* |
| **Version 3:**  not invasive,  adherence,  non-serious | *[timepoint 1 starts here]*  The doctor takes a moment to consult the computer program.  Doctor: “It’s as I suspected: IBS is by far the most likely cause of your symptoms. However, the computer program has flagged some alternative possibilities as well. Most are benign but one is potentially serious. The computer program suggests that we do a stool sample test, to investigate a potentially serious cause. This would involve collecting a small amount of poo in a pot (you can do this yourself at home), which we would send to the lab for analysis. Now, as I said, IBS is far more likely, but I still think it’s important to rule out anything sinister.” The doctor hands you the stool-collection kit and asks you to return a sample to the practice ASAP. The doctor also gives you some dietary advice and prescribes medication for the pain and constipation. You thank the doctor and leave the consultation room.  *[timepoint 1 measures taken here]*  *[timepoint 2 starts here]*  Please imagine that this is what happens after your GP appointment:  You collect a stool sample and return it to the practice. You also make the dietary changes that the doctor suggested and take the medication prescribed. The pain and constipation persist for about a week. At the start of the second week, the GP rings to discuss the results of your stool test: they are normal (no suggestion of a serious cause). Over the next few days, the pain and constipation begin to subside. They resolve completely within a week, so no further action is needed.  *[timepoint 2 measures taken here]* |
| **Version 4:**  invasive,  adherence,  non-serious | *[timepoint 1 starts here]*  The doctor takes a moment to consult the computer program.  Doctor: “It’s as I suspected: IBS is by far the most likely cause of your symptoms. However, the computer program has flagged some alternative possibilities as well. Most are benign but one is potentially serious. The computer program suggests that we do a colonoscopy, to investigate a potentially serious cause. A colonoscopy is a hospital procedure, whereby a thin tube (with a camera inside) is passed into your bottom to look for abnormalities*.* This can be very uncomfortable and there are some risks involved. For example, you may experience stomach cramps and anal bleeding after the procedure. In rare cases, you could get a small tear in your bowels, which may require surgery. Now, as I said, IBS is far more likely, but I still think it’s important to rule out anything sinister.” The doctor orders the colonoscopy, which will take place next week*.* The doctor also gives you some dietary advice and prescribes medication for the pain and constipation. You thank the doctor and leave the consultation room.  *[timepoint 1 measures taken here]*  *[timepoint 2 starts here]*  Please imagine that this is what happens after your GP appointment:  You make the dietary changes that the doctor suggested and take the medication prescribed. The pain and constipation persist for about a week. At the start of the second week, the colonoscopy takes place. It proceeds without any complications and the results are normal (no suggestion of a serious cause). Over the next few days, the pain and constipation begin to subside. They resolve completely within a week, so no further action is needed.  *[timepoint 2 measures taken here]* |
| **Version 5:**  not invasive,  no adherence,  serious | *[timepoint 1 starts here]*  The doctor takes a moment to consult the computer program.  Doctor: “It’s as I suspected: IBS is by far the most likely cause of your symptoms. However, the computer program has flagged some alternative possibilities as well. Most are benign but one is potentially serious. The computer program suggests that we do a stool sample test, to investigate a potentially serious cause. This would involve collecting a small amount of poo in a pot (you can do this yourself at home), which we would send to the lab for analysis. However, as I said, IBS is far more likely, so I don’t think that a stool test is necessary at this stage. I’ll spare you the hassle – it’s always an option down the line, if need be”. So, the doctor does not order the stool test, but gives you some dietary advice, prescribes medication for the pain and constipation, and tells you to come back in 2 weeks if they continue. You thank the doctor and leave the consultation room.  *[timepoint 1 measures taken here]*  *[timepoint 2 starts here]*  Please imagine that this is what happens after your GP appointment:  You make the dietary changes that the doctor suggested and take the medication prescribed. The pain and constipation persist for about a week. At the start of the second week, they get worse and you decide to go straight to A&E. The hospital doctors conduct a series of tests and identify an abnormal tissue growth known as a “polyp”. This is biopsied and found to be cancerous*.* Fortunately, it was caught early, so the chances of a successful treatment and recovery are high.  *[timepoint 2 measures taken here]* |
| **Version 6:**  invasive,  no adherence,  serious | *[timepoint 1 starts here]*  The doctor takes a moment to consult the computer program.  Doctor: “It’s as I suspected: IBS is by far the most likely cause of your symptoms. However, the computer program has flagged some alternative possibilities as well. Most are benign but one is potentially serious. The computer program suggests that we do a colonoscopy, to investigate a potentially serious cause. A colonoscopy is a hospital procedure, whereby a thin tube (with a camera inside) is passed into your bottom to look for abnormalities. This can be very uncomfortable and there are some risks involved. For example, you may experience stomach cramps and anal bleeding after the procedure. In rare cases, you could get a small tear in your bowels, which may require surgery. However, as I said, IBS is far more likely, so I don’t think that a colonoscopy is necessary at this stage. I’ll spare you the hassle – it’s always an option down the line, if need be.” So, the doctor does not order the colonoscopy but gives you some dietary advice, prescribes medication for the pain and constipation, and tells you to come back in 2 weeks if they continue. You thank the doctor and leave the consultation room.  *[timepoint 1 measures taken here]*  *[timepoint 2 starts here]*  Please imagine that this is what happens after your GP appointment:  You make the dietary changes that the doctor suggested and take the medication prescribed. The pain and constipation persist for about a week . At the start of the second week, they get worse and you decide to go straight to A&E. The hospital doctors conduct a series of tests and identify an abnormal tissue growth known as a “polyp”. This is biopsied and found to be cancerous*.* Fortunately, it was caught early, so the chances of a successful treatment and recovery are high.  *[timepoint 2 measures taken here]* |
| **Version 7:**  not invasive,  adherence,  serious | *[timepoint 1 starts here]*  The doctor takes a moment to consult the computer program.  Doctor: “It’s as I suspected: IBS is by far the most likely cause of your symptoms. However, the computer program has flagged some alternative possibilities as well. Most are benign but one is potentially serious. The computer program suggests that we do a stool sample test, to investigate a potentially serious cause. This would involve collecting a small amount of poo in a pot (you can do this yourself at home), which we would send to the lab for analysis. Now, as I said, IBS is far more likely, but I still think it’s important to rule out anything sinister.” The doctor hands you the stool-collection kit and asks you to return a sample to the practice ASAP. The doctor also gives you some dietary advice and prescribes medication for the pain and constipation. You thank the doctor and leave the consultation room.  *[timepoint 1 measures taken here]*  *[timepoint 2 starts here]*  Please imagine that this is what happens after your GP appointment:  You collect a stool sample and return it to the GP practice. You also make the dietary changes that the doctor suggested and take the medication prescribed. The pain and constipation persist for about a week. At the start of the second week, the GP rings to discuss the results of your stool test: a small amount of blood was identified in your stool and additional hospital tests are needed. At the hospital, the doctors conduct more tests and identify an abnormal tissue growth known as a “polyp”. This is biopsied and found to be cancerous*.* Fortunately, it was caught early, so the chances of a successful treatment and recovery are high.  *[timepoint 2 measures taken here]* |
| **Version 8:**  invasive, adherence, serious | *[timepoint 1 starts here]*  The doctor takes a moment to consult the computer program.  Doctor: “It’s as I suspected: IBS is by far the most likely cause of your symptoms. However, the computer program has flagged some alternative possibilities as well. Most are benign but one is potentially serious. The computer program suggests that we do a colonoscopy, to investigate a potentially serious cause. A colonoscopy is a hospital procedure, whereby a thin tube (with a camera inside) is passed into your bottom, to look for abnormalities*.* This can be very uncomfortable and there are some risks involved. For example, you may experience stomach cramps and anal bleeding after the procedure. In rare cases, you could get a small tear in your bowels, which may require surgery. Now, as I said, IBS is far more likely, but still I think it’s important to rule out anything sinister.” The doctor orders the colonoscopy, which will take place next week*.* The doctor also gives you some dietary advice and prescribes medication for the pain and constipation. You thank the doctor and leave the consultation room.  *[timepoint 1 measures taken here]*  *[timepoint 2 starts here]*  Please imagine that this is what happens after your GP appointment:  You make the dietary changes that the doctor suggested and take the medication prescribed. The pain and constipation persist for about a week. At the start of the second week, the colonoscopy takes place. It proceeds without any complications. The results show an abnormal tissue growth known as a “polyp”. This is biopsied and found to be cancerous*.* Fortunately, it was caught early, so the chances of a successful treatment and recovery are high.  *[timepoint 2 measures taken here]* |

**SM2: Demographic questions**

1. What is your age in years?

___ years

1. What is your gender?

- Female
- Male
- Non-binary
- Prefer not to say

1. What is your ethnic group?

Please choose one option that best describes your ethnic group or background:

- **White**
- **Mixed/Multiple ethnic groups**
- **Asian/Asian British**
- **Black/ African/Caribbean/Black British**
- **Other ethnic group**

1. What is your highest educational qualification?

- Less than high school
- High school graduate
- Undergraduate university degree (e.g., Bachelor’s)
- Postgraduate university degree (e.g., Masters, Doctorate)

**SM3: Trust in Physicians Scale^1^**

Please answer the following questions in regards to your regular GP/GP practice:

1. I doubt that my doctor really cares about me as a person.^a^
2. My doctor is usually considerate of my needs and puts them first.
3. I trust my doctor so much I always try to follow his/her advice.
4. If my doctor tells me something is so, then it must be true.
5. I sometimes distrust my doctor’s opinion and would like a second one.^a^
6. I trust my doctor’s judgments about my medical care.
7. I feel my doctor does not do everything he/she should for my medical care.^a^
8. I trust my doctor to put my medical needs above all other considerations when treating my medical problems.
9. My doctor is a real expert in taking care of medical problems like mine.
10. I trust my doctor to tell me if a mistake was made about my treatment.
11. I sometimes worry that my doctor may not keep the information we discuss totally private.^a^

Participants will be asked to indicate their agreement with each item on a 5-point scale (1=*strongly disagree* to 5=*strongly agree*).

^a^ = negatively worded item

**SM4: Health Regulatory Focus questionnaire^2^**

1. I frequently imagine how I can achieve a state of “ideal health”.
2. I think of good health as a key to a happy life.
3. Doing healthy things gives me a sense of accomplishment.
4. When I engage in healthy behaviours, I am pleased with myself.
5. I would do anything to maintain a good, healthy body.
6. I admire people who do things that make them very healthy.
7. I often worry that I am not doing the best I can to improve my health.
8. I often imagine myself being ill in the future.
9. I am anxious that I am not following through on my obligations and being as responsible as I should about taking care of my health.
10. When I see people who are very sick because they did not take care of their health, I get scared thinking that could be me in the future.
11. I often worry about not feeling as healthy as I used to be.
12. Thinking about my health usually makes me worry.

Respondents are asked to indicate their agreement with each item on a 7-point scale (1=*strongly disagree* to 7=*strongly agree*).

**SM5: Single-item Maximizer-Minimizer Elicitation** **Question (MM1)^3^**

Sometimes, medical action is clearly necessary, and sometimes it is clearly NOT necessary. Other times, reasonable people differ in their beliefs about whether medical action is needed.

In situations where it’s not clear, do you tend to lean towards **taking action** or do you lean towards **waiting and seeing** if action is needed?

**Importantly, there is no “right” way to be.**

Please answer on the 1-6 scale below:


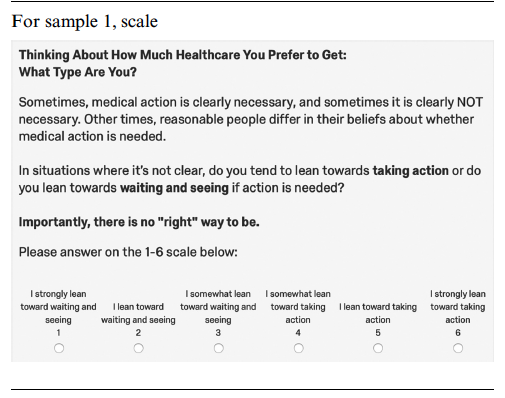


**SM6: Health-related questions**

1. How physically fit are you, compared to the average person of your age?

- More fit
- Equally fit
- Less fit

1. Do you suffer from chronic illness?

- Yes
- No

1. How many GP consultations have you attended in the past 12 months (remote or in-person)?

- 0-4
- 5-9
- 10-14
- 15-19
- 20+

1. Why do you think GPs use computers during clinical consultations? Please tick all that apply (or none, if none apply):

- For reimbursement purposes
- To make sure they record my information correctly and completely
- To make sure that they remember what happened in my past consultations
- To make sure that they do not miss anything, e.g. screening tests, that are due.
- To avoid talking directly to me
- To sell my data to pharmaceutical companies
- Other, please explain:

**SM7: Regression models with individual differences, status of health, and demographic variables**

Table SM7­­­ displays the results of exploratory regression models that included the following covariates: worry, attitudes toward health and healthcare (Health Regulatory Focus, Trust In Physicians, Minimization-Maximization), state of health (fitness, chronic illness, number of consultations in the last year), and demographics (age, gender^[[1]](#footnote-1)^*). Greater health regulatory focus (HRF) and trust in physicians (TIP) were both associated with significantly more favourable ratings on all three measures (satisfaction, recommendation, and DDA use). No associations were detected with Minimization-Maximization (MM1). Female respondents gave significantly lower ratings on all measures than male respondents. No associations with respondent age were detected.

|  | **Timepoint 1** | | | **Timepoint 2** | | |
| --- | --- | --- | --- | --- | --- | --- |
|  | Satisfaction  *b* [95% CI]  p | Recommendation  *b* [95% CI]  p | DDA use  *b* [95% CI]  p | Satisfaction  *b* [95% CI]  p | Recommendation  *b* [95% CI]  p | DDA use  *b* [95% CI]  p |
| Adherence | -3.09  [-13.95, 7.77]  *p*=0.577 | -0.11  [-0.47, 0.26]  *p*=0.565 | -0.02  [-0.42, 0.38]  *p*=0.916 | -0.86  [-13.19, 11.48]  *p*=0.892 | 0.01  [-0.40, 0.41]  *p*=0.975 | 0.24  [-0.20, 0.68]  *p*=0.278 |
| Invasiveness | -0.32  [-11.22, 10.59]  *p*=0.955 | -0.08  [-0.44, 0.29]  *p*=0.686 | -0.25  [-0.65, 0.15]  *p*=0.221 | 5.05  [-7.45,17.55]  *p*=0.428 | 0.16  [-0.25, 0.57]  *p*=0.435 | -0.19  [-0.64, 0.25]  *p*=0.397 |
| Severity | -- | -- | -- | -16.37  [-28.95, -3.79]  *p*=0.011 | -0.49  [-0.91, -0.08]  *p*=0.020 | -0.17  [-0.61, 0.28]  *p*=0.467 |
| Adherence x Severity | -- | -- | -- | 14.29  [4.87, 23.71]  *p*=0.003 | 0.55  [0.24, 0.86]  *p*<0.001 | 0.40  [0.06, 0.73]  *p*=0.020 |
| Invasiveness x Severity | -- | -- | -- | 4.33  [-5.16, 13.81]  *p*=0.371 | 0.16  [-0.15, 0.48]  *p*=0.303 | 0.18  [-0.15, 0.52]  *p*=0.287 |
| Adherence x Invasiveness | -4.72  [-11.01, 1.56]  *p*=0.141 | -0.13  [-0.34, 0.08]  *p*=0.225 | 0.14  [-0.09, 0.37]  *p*=0.230 | -4.41  [-13.77, 4.95]  *p*=0.355 | -0.16  [-0.47, 0.15]  *p*=0.298 | 0.23  [-0.10, 0.56]  *p*=0.171 |
| Adherence x Invasiveness x Severity | -- | -- | -- | -2.66  [-15.98, 10.66]  *p*=0.695 | -0.02  [-0.45, 0.42]  *p*=0.943 | -0.20  [-0.67, 0.27]  *p*=0.408 |
| Worry | -9.92  [-13.32, -6.52]  *p*<0.001 | -0.29  [-0.40, -0.17]  *p*<0.001 | 0.07  [-0.05, 0.20]  *p*=0.268 | -7.23  [-11.35, -3.10]  *p*=0.001 | -0.09  [-0.23, 0.04]  *p*=0.178 | 0.11  [-0.04, 0.25]  *p*=0.151 |
| Worry x Adherence | 7.73  [3.87, 11.60]  *p*<0.001 | 0.24  [0.11, 0.37]  *p*<0.001 | 0.02  [-0.13, 0.16]  *p*=0.819 | 5.60  [1.50, 9.70]  *p*=0.007 | 0.16  [0.02, 0.29]  *p*=0.023 | -0.11  [-0.26, 0.03]  *p*=0.134 |
| Worry x Invasiveness | 3.05  [-.77, 6.88]  *p*=0.118 | 0.10  [-0.03, 0.23]  *p*=0.123 | 0.05  [-0.09, 0.19]  *p*=0.445 | 0.42  [-3.64, 4.47]  *p*=0.841 | -0.01  [-0.14, 0.13]  *p*=0.900 | 0.03  [-0.11, 0.18]  *p*=0.646 |
| Worry x Severity | -- | -- | -- | 0.04  [-4.01, 4.08]  *p*=0.987 | -0.04  [-0.18, 0.09]  *p*=0.513 | 0.01  [-0.14, 0.15]  *p*=0.930 |
| Health Regulatory Focus (HRF) | 0.30  [0.16, 0.45]  *p*<0.001 | 0.01  [0.01, 0.02]  *p*<0.001 | 0.01  [0.01, 0.02]  *p*<0.001 | 0.31  [0.16, 0.47]  *p*<0.001 | 0.01  [0.01, 0.02]  *p*<0.001 | 0.02  [0.01, 0.02]  *p*<0.001 |
| Trust in Physicians (TIP) | 0.97  [0.76, 1.18]  *p*<0.001 | 0.03  [0.03, 0.04]  *p*<0.001 | 0.01  [0.00, 0.02]  *p*=0.010 | 1.17  [0.95, 1.39]  *p*<0.001 | 0.04  [0.03, 0.05]  *p*<0.001 | 0.02  [0.01, 0.02]  *p*<0.001 |
| Minimization-Maximization (MM1) | 0.21  [-0.92, 1.34]  *p*=0.713 | 0.01  [-0.02, 0.05]  *p*=0.480 | 0.06  [0.02, 0.10]  *p*=0.003 | 0.39  [-0.81, 1.59]  *p*=0.523 | -0.00  [-0.04, 0.04]  *p*=0.940 | 0.04  [-0.01, 0.08]  *p*=0.089 |
| Fitness | 1.71  [-0.63, 4.05]  *p*=0.152 | 0.08  [-0.003, 0.15]  *p*=0.060 | 0.07  [-0.02, 0.16]  *p*=0.105 | 1.83  [-0.65, 4.31]  *p*=0.147 | 0.05  [-0.04, 0.13]  *p*=0.263 | 0.10  [0.02, 0.19]  *p*=0.021 |
| Chronic illness | 0.72  [-3.20, 4.63]  *p*=0.720 | 0.06  [-0.07, 0.19]  *p*=0.344 | -0.01  [-0.15, 0.14]  *p*=0.950 | 1.36  [-2.80, 5.52]  *p*=0.521 | 0.09  [-0.04, 0.23]  *p*=0.181 | 0.04  [-0.11, 0.18]  *p*=0.641 |
| Consultations in the last year | 0.32  [-2.02, 2.65]  *p*=0.791 | 0.02  [-0.06, 0.10]  *p*=0.567 | 0.06  [-0.03, 0.14]  *p*=0.186 | 0.41  [-2.07, 2.88]  *p*=0.746 | -0.05  [-0.13, 0.03]  *p*=0.201 | 0.04  [-0.05, 0.13]  *p*=0.402 |
| Age | 0.11  [-0.01, 0.22]  *p*=0.063 | 0.00  [-0.00, 0.01]  *p*=0.062 | 0.00  [-0.00, 0.01]  *p*=0.347 | 0.06  [-0.07, 0.18]  *p*=0.367 | 0.00  [-0.00, 0.01]  *p*=0.568 | 0.01  [0.00, 0.01]  *p*=0.031 |
| Gender | -5.90  [-9.26, -2.55]  *p*=0.001 | -0.11  [-0.22, 0.00]  *p*=0.051 | -0.17  [-0.30, -0.05]  *p*=0.005 | -3.53  [-7.09, 0.04]  *p*=0.052 | -0.07  [-0.19, 0.05]  *p*=0.231 | -0.15  [-0.28, -0.03]  *p*=0.017 |

**Table SM7.** Regression models with individual differences, status of health, and demographic variables.

- *Cells contain regression coefficients (b), 95% confidence intervals, and p values.*
- ***Invasiveness****,* ***Adherence*** *and* ***Severity*** *were coded 0=not invasive/no adherence/not serious, 1=invasive/adherence/serious.* ***Worry*** *was coded 1=not at all worried, 2=a little bit worried, 3=moderately worried, 4=very worried.* ***Fitness*** *was coded 1=less fit, 2=equally fit, 3=more fit.* ***Chronic illness*** *was coded 0=absent, 1=present.* ***Consultations in the last year*** *was coded* *1=0-4, 2=5-9, 3=10-14, 4=15-19, 5=20+.* ***Gender*** *was coded 0=male, 1=female.*

**SM8: Results of planned analysis for “satisfaction with doctor”**

The mean was 66.9 (*SD*=25.4) at timepoint 1 and 68.2 (*SD*=27.8) at timepoint 2. The results of the planned regression models are tabulated below.

|  | **Timepoint 1**  *b* [95% CI], *p* | **Timepoint 2**  *b* [95% CI], *p* |
| --- | --- | --- |
| Adherence | 19.3  [14.4, 24.2]  *p*<0.001 | 16.6  [9.5, 23.8]  *p*<0.001 |
| Invasiveness | 9.6  [4.6, 14.6]  *p*<0.001 | 9.1  [1.8, 16.5]  *p*=0.015 |
| Severity | -- | -17.5  [-24.8, -10.2]  *p*<0.001 |
| Severity x Adherence | -- | 14.9  [4.6, 25.3]  *p*=0.005 |
| Severity x Invasiveness | -- | 1.7  [-8.7, 12.2]  *p*=0.743 |
| Adherence x Invasiveness | -6.5  [-13.4, 0.5]  *p*=0.068 | -9.1  [-19.3, 1.2]  *p*=0.082 |
| Adherence x Invasiveness x Severity | -- | 3.00  [-11.6, 17.6]  *p*=0.686 |
| (Constant) | 53.8  [50.3, 57.3]  *p*<0.001 | 61.5  [56.4, 66.6]  *p*<0.001 |

**Table SM8.** Effect of the manipulated factors on interim (t1) and final (t2) measures of satisfaction with the doctor.

**SM9: Results of planned analysis for “satisfaction with the consultation”**

The mean was 66.8 (*SD*=26.6) at timepoint 1 and 68.3 (*SD*=28.4) at timepoint 2. The results of the planned regression models are presented below.

|  | **Timepoint 1**  *b* [95% CI], *p* | **Timepoint 2**  *b* [95% CI], *p* |
| --- | --- | --- |
| Adherence | 21.1  [15.9, 26.2]  *p*<0.001 | 17.7  [10.3, 25.1]  *p*<0.001 |
| Invasiveness | 8.2  [3.0, 13.3]  *p*=0.002 | 9.4  [1.8, 17.0]  *p*=0.015 |
| Severity | -- | -15.2  [-22.8, -7.7]  *p*<0.001 |
| Severity x Adherence | -- | 12.6  [1.9, 23.2]  *p*=0.021 |
| Severity x Invasiveness | -- | -0.90  [-11.6, 9.8]  *p*=0.869 |
| Adherence x Invasiveness | -5.6  [-12.8, 1.7]  *p*=0.131 | -8.0  [-18.5, 2.5]  *p*=0.134 |
| Adherence x Invasiveness x Severity | -- | 3.4  [-11.6, 18.4]  *p*=0.659 |
| (Constant) | 53.4  [49.8, 57.0]  *p*<0.001 | 60.8  [55.5, 66.1]  *p*<0.001 |

**Table SM9.** Effect of the manipulated factors on interim (t1) and final (t2) satisfaction with the consultation.

**SM10: Why do doctors use computers in the consultation? Number and proportion of participants that selected each reason**

| **Why do doctors use computers in the consultation?** | ***N*** | **%** |
| --- | --- | --- |
| For reimbursement purposes | 67 | 9.2% |
| To make sure they record my information correctly and completely | 515 | 70.5% |
| To make sure that they remember what happened in my past consultations | 448 | 61.4% |
| To make sure that they do not miss anything, e.g., screening tests that are due | 417 | 57.1% |
| To avoid talking directly to me | 64 | 8.8% |
| To sell my data to pharmaceutical companies | 33 | 4.5% |
| Other | 9 | 1.2% |

NB. Participants could select multiple reasons; therefore, percentages do not sum to 100.

References

1. Anderson LA and Dedrick RF. Development of the Trust in Physician scale: a measure to assess interpersonal trust in patient-physician relationships. *Psychological reports* 1990; 67: 1091-1100.

2. Ferrer RA, Lipkus IM, Cerully JL, et al. Developing a scale to assess health regulatory focus. *Social Science & Medicine* 2017; 195: 50-60. DOI: <https://doi.org/10.1016/j.socscimed.2017.10.029>.

3. Scherer LD and Zikmund-Fisher BJ. Eliciting Medical Maximizing-Minimizing Preferences with a Single Question: Development and Validation of the MM1. *Medical Decision Making* 2020; 40: 545-550. DOI: 10.1177/0272989x20927700.

1. * The preregistered analysis included education and ethnicity as well; these were not carried out due to very small numbers in some of the categories, which could produce misleading results. [↑](#footnote-ref-1)
